# Multisensory perception constrains the formation of object categories: a review of evidence from sensory-driven and predictive processes on categorical decisions

**DOI:** 10.1098/rstb.2022.0342

**Published:** 2023-09-25

**Authors:** F. N. Newell, E. McKenna, M. A. Seveso, I. Devine, F. Alahmad, R. J. Hirst, A. O'Dowd

**Affiliations:** School of Psychology and Institute of Neuroscience, Trinity College Dublin, College Green, Dublin D02 PN40, Ireland

**Keywords:** multisensory, object perception, object categorization, categorical perception, statistical learning, lifespan

## Abstract

Although object categorization is a fundamental cognitive ability, it is also a complex process going beyond the perception and organization of sensory stimulation. Here we review existing evidence about how the human brain acquires and organizes multisensory inputs into object representations that may lead to conceptual knowledge in memory. We first focus on evidence for two processes on object perception, multisensory integration of redundant information (e.g. seeing and feeling a shape) and crossmodal, statistical learning of complementary information (e.g. the ‘moo’ sound of a cow and its visual shape). For both processes, the importance attributed to each sensory input in constructing a multisensory representation of an object depends on the working range of the specific sensory modality, the relative reliability or distinctiveness of the encoded information and top-down predictions. Moreover, apart from sensory-driven influences on perception, the acquisition of featural information across modalities can affect semantic memory and, in turn, influence category decisions. In sum, we argue that both multisensory processes independently constrain the formation of object categories across the lifespan, possibly through early and late integration mechanisms, respectively, to allow us to efficiently achieve the everyday, but remarkable, ability of recognizing objects.

This article is part of the theme issue ‘Decision and control processes in multisensory perception’.

## 1. Introduction

Object categorization is a fundamental cognitive ability that allows us to efficiently recognize and interact with the content of our environment despite variability in the encoded object properties from one instance, or from one example, to the next. The processes underpinning category formation are thought to be mediated by both sensory-driven information and prior knowledge from memory, although how object categories emerge based on multisensory features is poorly understood. Indeed, direct links from categorization to perceptual processes have been disputed. For example, one proposal suggests that the mechanisms that support memory processes are shared with those underpinning perception [1,2], thus the formation of object categories in memory may be understood by investigating the nature of multisensory object perception. By contrast, others have argued against the proposal for shared cognitive and perceptual processing and propose a more modular approach such that the processes underpinning object perception and memory may be more separable [3,4]. In this article, we first review the evidence for how object perception is achieved based on two distinct processes, multisensory integration or learned crossmodal associations across features. We then ask how each of these perceptual mechanisms might form the basis of object categories in memory. In addition, investigations of the neural underpinnings of multisensory processes, and consequent influence on object perception, have provided important insights into the formation of categories. Furthermore, recent studies have revealed how development and ageing affect multisensory integration in distinct ways, mainly due to changes in the structure and function of sensory and neural systems. Given these differences, we consider how the formation of stable, multisensory representations of objects in memory and consequent categorical decisions may occur across the lifespan. Based on this knowledge, we propose a distinction between early (sensory-driven) and late (learned associations) interactions across modalities and show how each supports efficient object categorization. We argue that recent insights into multisensory perceptual processes help provide a rich framework for understanding the formation of multisensory object categories in memory.

In the past, our knowledge of how objects are perceived was mainly based on visual processing alone. This body of research revealed that to achieve perception, object information is processed in increasing levels of complexity along the ventral, or so-called ‘what’, pathway in the temporal lobe, from low-level feature processing to object shape representations that are largely invariant to incidental changes in the environment [5–8]. Solutions to the problem of variability in an object's encoded image (e.g. blurring or unfamiliar viewpoint) have been met by theoretical proposals based on structural descriptions [9,10] or image-based [11] theories, with each assuming different levels of object specificity. These proposals can be extended to explain object categorization based on shared structures or perceptual similarity [12–14], respectively. While successful categorization requires generalization across all possible visual features [15], a distinction between features of individual objects from within the same category is required for object recognition. Consistent with this, there is evidence for a transition from object-based information to category processing along the posterior to anterior axis of the temporal lobe [16]. For example, there is strong evidence for shape selectivity in the lateral occipital complex (LOC, [17]) and motion selectivity in superior temporal sulcus (STS, [18]), with each representing object-specific information that is sensory- or perceptually based. Other evidence for age-related changes in activity in the LOC during visual object processing [19] is also consistent with a differentiation of object representations in the ventral visual stream [20,21]. By contrast, higher regions of the cortex, including the prefrontal cortex, represent the cognitive aspects of categorization such as decisions on category membership and adaptability to task set [22]. Indeed, comparative studies provide evidence for a distinction between the roles of temporal and frontal regions in the perceptual and cognitive aspects of categorization, respectively [23]. Object perceptual information therefore culminates in memory structures within the medial (MTL) and anterior temporal lobe (ATL)—specifically, the perirhinal cortex (PRC; e.g. [24,25])—for the formation of categories that can, in turn, influence object-based processing in ventral areas [26]. The evidence for a distribution of information from object-selective regions within the temporal lobe to later regions within the ATL appears to support many aspects of object categorization, at least in the visual domain.

However, it is clear that individual objects can be perceived by a variety of features other than shape, such as sound, weight, surface texture and even taste or smell, and such multisensory representations likely lead to more robust object perception [27]. However, to date, relatively few studies have systematically examined the contribution of information from other sensory systems to the formation or maintenance of categories in memory. In those few exceptions, while interesting proposals have been put forward for how featural information (or memory traces) can be integrated across modalities for object categorization (see e.g. [28–30]), these often do not sufficiently account for the various ways in which multisensory (behavioural or neural) processes underpin the perception of objects, nor explain the specific processes involved in merging crossmodal object information in memory, which can be affected by sensory or perceptual changes across the lifespan.

## 2. How does multisensory information contribute to the perception of objects?

There are inherent differences in information processing across the senses, including from one moment to the next or due to longer age-related changes, which render the integration of this information particularly complex. For example, there may be differences in intensity profiles, or noise in the encoded information, or the receptive field size of activated neurons may differ across sensory systems. To date, a number of solutions have been proposed for how multisensory integration may be achieved for object perception that have both biological and behavioural relevance. For example, the integration of multisensory inputs is thought to be guided by spatial and temporal coherence, such that features or events that occur together in time and/or location are readily integrated [31]. The consequent integration of crossmodal cues into a coherent percept facilitates perceptual decisions relative to unisensory inputs alone [32]. The benefits of integrating information across the senses is demonstrated with enhancement in activation at the single neuron level [33] and in regional activations in the brain [34–36], as well as improved behavioural performance (e.g. [37]). Importantly, there is consistent evidence that multisensory integration enhances perception and influences perceptual decisions in a manner that is not predicted by probability summation of either unisensory, such as two visual cues, or crossmodal inputs alone [33,38–41].

In essence, multisensory information can influence perceptual decision processes either by enhancing the reliability of the integrated percept relative to the individual sensory cues or by facilitating learning of crossmodal associations. In other words, sensory information about an object can be either redundant across modalities (i.e. shared stimulus properties, such as the shape of an object encoded through vision and haptics) or complementary (e.g. the colour and weight of an object), and object information is either merged based on sensory reliability [42] or based on incidental or statistical learning of crossmodal associations from the environment [43]. Furthermore, prior knowledge can facilitate the binding of redundant cues due to the allocation of selective attention during encoding [44]. These differences—that is, whether crossmodal information is integrated based on cue redundancy or statistical learning—suggest that there may be separate mechanisms underpinning the formation of multisensory categories, and that each may be attributed to early or late integration in the brain (as we discuss in detail in §5).

A number of statistical or Bayesian models of behavioural performance have suggested that the brain combines information from different senses in an optimal manner, based particularly on the inferred causality [45] or reliability of the information from each modality [46]. For example, Bayesian models of multisensory integration typically consider both bottom-up and top-down influences in perceptual decisions, such as information reliability and causal inference from the ‘coupling prior’ [32,47]. A coupling prior is the prior probability that the co-occurring information or signals from different sensory modalities may have a common cause. As such, information from multiple senses can be integrated based on the respective reliability of each input in a bottom-up manner or on the inferred causality based on prior knowledge to construct a coherent representation of the object [27,42]. An example of a possible source of reliability in sensory information for object perception is the relative salience, or distinctiveness, of features across modalities. Thus, a distinctive feature in one modality (e.g. intensity, or high frequency of occurrence) may have a greater relative weighting across modalities and aid in the subsequent perception of the object [48,49]. For example, the intensity of coffee may be more reliably perceived by its aroma than by seeing its dark liquid.

Predictive coding models posit that the brain maintains an internal model, or best estimate (priors or templates), of the environment that is updated, not by the sensory inputs alone, but by the error between the expected and actual sensory inputs (i.e. forward connections) [50]. Thus, a combination of internal predictions and sensory signals is merged into the most probable representation of the stimulus. This internal representation provides top-down estimates of the environment to minimize uncertainty, and sensory information either confirms these estimates or produces an error signal that leads to the updating of the internal model to affect multisensory perceptual decisions [51]. Any incongruency between the sensory information and the internal prediction, which leads to the adjustment of the model, is considered a bottom-up process. By contrast, the top-down modulation of sensory processing by internal estimations from prior representations is thought to be mediated by one of a number of processes including selective attention, context-dependent regularities, action and working memory, with the most relevant information being prioritized in a task- or stimulus-specific manner [29,52].

To date, processes such as causal inference or predictive coding have mainly been proposed to explain cross-sensory interactions for perception and have not adequately been considered in models of the formation of multisensory object categories in memory, despite the relevance. Given the increasing knowledge of multisensory interactions in the brain, we argue that there is a timely need for an extension of these models. For example, traditionally it was assumed that multisensory integration occurred late in information processing, underpinned by activations in association cortex or beyond in neural structures supporting associative learning and memory. However, evidence for crossmodal interactions in primary sensory regions of the brain, revealing plasticity changes to multisensory inputs at all stages of brain processing, is now overwhelming [53]. Indeed, the past few decades have witnessed growing evidence for multisensory interactions within primary sensory regions of the brain, including activations in the visual cortex to auditory and somatosensory inputs [54] and responses in primary auditory cortex to visual inputs [55], suggesting that cross-sensory inputs moderate early stages of information processing. Multisensory interactions have also been reported along temporal regions of the brain, with evidence based on single neuron recordings [56–58] to neuroimaging studies of the human brain [59,60]. More pertinently, crossmodal interactions specific to object perception have been found in regions of the temporal lobe that were once thought to be selective for visual-only processing of objects [61,62]. For example, both tactile object exploration [61] and tactile–auditory shape learning [63] lead to an increase in activation in the LOC, and tactile encoding of moving objects leads to activation in visual area of the brain referred to as the human middle temporal cortex (hMT) [64]. Evidence that the categorical organization and selectivity of the visual temporal cortex are maintained even in the absence of visual experience across development [65–68] provides further support to the idea of modality-independent information processing. Indeed, some argue that these activations along the ventral stream of the temporal lobe may be influenced more by the task-specific nature of the encoded information (e.g. spatial or temporal) rather than its sensory origin [69–71].

In addition to integrated sensory inputs, an important role for incidental or statistical learning of crossmodal features in perceptual decisions has long been established [72]. As such, whenever (uncorrelated) crossmodal sensory features have a high probability of appearing together in the environment, which Barlow referred to as ‘suspicious coincidences’ [73,74], these cues will be associated in memory such that the presentation of one should predict the presentation of the other to enhance perception [75]. Commonly learned statistical regularities, such as visuo-spatial elevation and auditory pitch [76], are sometimes referred to as ‘crossmodal correspondences’ [77] and are implicitly acquired (i.e. without awareness) from repeated exposure to these coincident cues in the environment. Investigations of object perception suggest that these learned crossmodal associations facilitate subsequent object perception and recognition, even when information from only one modality is available [78]. For example, unimodal object perception is enhanced by the prior presentation of semantically congruent (e.g. image of a helicopter and sound of a propeller) compared to incongruent (e.g. image of a cat and sound of an owl) crossmodal features [79]. These findings suggest that prior expectations, triggered by semantic associations, can facilitate the subsequent perception of objects in any modality [80]. Indeed, this facilitation is likely to occur during a later, post-sensory, stage in information processing. For example, using EEG, Franzen *et al*. [81] reported evidence that the visual perception of familiar objects, such as faces or cars, is facilitated by semantically congruent sounds (speech or engine noises, respectively) at a late stage associated with decision-related processing.

Taken together, the results of investigations into the multisensory perception of objects suggest that the reliability of sensory information and learned associations across modalities can directly influence perceptual decisions [82]. These processes are, in fact, not likely to be mutually exclusive since sensory information about familiar objects can vary in its reliability (e.g. incidentally or with developmental changes), and the predictive nature of an object's features may differ across modalities. Indeed, research into multisensory object perception suggests that categories may be shaped by all relevant information available, often in a flexible way according to task demands. To date, however, investigations into the formation of object categories across multiple features [83] or dimensions [84] have mainly been based on visual processing [85], although some recent models refer to crossmodal feature integration at late stages aligned with action or motor processes [86]. However, as discussed earlier, late feature integration does not sufficiently capture the breadth of processes involved in multisensory integration, nor the specific benefits arising from early integration that can have quantitative [82,87] as well as qualitative or phenomenological [88–90] effects on perception. The recent increase in knowledge of multisensory perceptual processes, from neuronal interactions to behavioural performance, can be leveraged towards a better understanding of multisensory interactions underpinning object categorization and, in turn, provide more ecological models of how the brain processes and organizes sensory information from the environment.

## 3. How are multisensory object categories formed in memory?

In parallel with our increasing knowledge of category organization of visual regions of the human brain (e.g. [5]), cognitive models have provided an important theoretical framework for understanding how sensory information is organized in memory to allow us to perceive and recognize objects [91–93]. Compelling behavioural evidence for a fine-grained taxonomy of conceptual knowledge has been provided, which further supports cognitive efficiency by allowing for novel exemplars or objects to be recognized as members of an existing, learned object category. For example, in a series of classic studies, Rosch *et al*. [94] provided evidence that objects are represented in memory according to a taxonomy from abstract to specific. According to this approach, information is organized into hierarchical networks of object knowledge, from subordinate (e.g. Labrador), basic level (e.g. dog) and superordinate (e.g. domesticated animal) levels of classification. Their research suggested an advantage for basic level categorization [94] as basic level categories were identified as the entry point for visual categorization. Other evidence supports the idea that categorization at various levels of abstraction has a distinct time course: basic level categorization is generally fastest although an advantage for superordinate processing during rapid categorization tasks can also be found [95].

In contrast with a layered, taxonomic organization of object categories, others have argued for a more bottom-up approach. Accordingly, it is proposed that category formation is grounded in perception [96] such that objects are recognized based on their perceptual similarity to internal representations in memory. Thus, object recognition performance is facilitated when an object's image is most similar to the representation of an exemplar from a previously learned category [11,12]. Moreover, it is argued that inter-object similarity also forms the basis of object categories, which allows for the efficient recognition of familiar as well as novel exemplars by perceiving the objects from within a category as perceptually equivalent and those beyond the category boundary as distinct [13,14]. Indeed, studies of human perceptual processing have revealed that this organization of information into categories has a direct effect on how objects are perceived. This phenomenon is known as ‘categorical perception’ (CP) and is experimentally revealed when two stimuli that belong to the same category are more perceptually similar than two that belong to different categories that are perceptually more discriminable, despite these object pairs being equally distant in units of physical measurement [97,98]. CP is thought to be a fundamental process as well as being a universal organizing principle for information processing: evidence of CP has been found to sounds in crickets [99], birds [100] and in non-human primates [101].

Top-down predictions of object categorization acquired through associative or statistical learning also help provide insight into how complex information is processed to form categories [102]. For example, the probability of co-occurrence of certain features in the environment may be a source of information that reliably predicts category membership [75]. Indeed, evidence for statistical learning of crossmodal associations in adults [103] as well as in children [104] suggests that these learned regularities are a rich source of information and likely to influence the formation of object categories in memory [105]. Although previous studies suggested that statistical learning involved domain-general mechanisms, some recent findings suggest that learning is constrained by the working limits of each sensory system (e.g. the processing of temporal information is enhanced in the auditory system whereas vision is optimized for spatial information: e.g. [106] (see [102] for a discussion)). As such, the efficiency by which associations between crossmodal object features are learned for the purpose of categorization is likely to depend on perceptual processes. Moreover, the nature of the learned information, or the regularity of the crossmodal associations, may affect the underlying structure of the categories or perceptual decisions regarding category membership. For example, if a visual feature (e.g. elevation) is perfectly correlated with an auditory feature (e.g. pitch) then it is likely that one unisensory feature may suffice for categorization. On the other hand, a partial correlation may lead to a more complex category structure, with category decisions necessarily dependent on multiple sources of information [107]. The precise mechanisms by which top-down knowledge and multisensory-driven processes interact to affect behaviour are currently not well understood.

Nevertheless, converging evidence from neurophysiological and neuroimaging studies can provide important insights into the top-down processes that facilitate the formation of categories and highlight their adaptive properties [108,109]. For example, in a landmark study, Freedman *et al*. [110] reported object category effects in a population of neurons in the lateral prefrontal cortex. These neurons selectively responded to learned categories of object stimuli (computer-generated or morphed images of cats and dogs that varied in shape, e.g. 60% cat and 40% dog) with a defined category decision boundary and could readily adapt to the relearning of category membership. Other studies have highlighted plasticity changes in the brain following perceptual learning of objects, particularly changes in ‘neural tuning’ in sensory [111–113] as well as prefrontal regions (see [114]), often in a task-dependent manner [115,116]. Similarly, results from several neuroimaging studies have elucidated the neural processes involved in CP in the human brain [117,118] and suggest that interactions between the temporal and prefrontal cortices as well as other brain regions may act as a network in the formation of object categories [119,120]. In particular, top-down feedback may subsequently hone neuronal selectivity in temporal regions towards diagnostic or task-relevant features for object categorization or discrimination [121,122] and render perception more efficient [123].

Based on the evidence discussed above, it is reasonable to assume that investigations of multisensory processes may reveal a similar organization of category-specific responses within object-selective regions of the brain, although how this complex process emerges across modalities is currently unclear. Nevertheless, some studies provide important insights into the location of these interactions in the brain [124]. The PRC, in particular, has been proposed as the cortical region subserving semantic knowledge of objects [125] (see also [126]) and has been found to be involved in binding object features across modalities. For example, Taylor *et al*. [127] reported activation in the PRC that distinguished between semantically congruent (e.g. an image of a cow and sound of ‘moo’) and incongruent audio-visual information and mirrored these findings in patients with lesions in brain regions that included the PRC who were impaired in these tasks. Importantly, no such effects of semantic congruency in lower brain regions that are known to have multisensory functions, such as the STS, were found. Similarly, visuo-tactile matching of novel object shapes (i.e. without prior semantic knowledge) is also associated with greater activation in the PRC than within-modality shape matching, particularly for shape information that is congruent (i.e. from the same object) across modalities [128]. Together these findings suggest that the PRC is involved in multisensory representations of objects, and that object features may be integrated into the representation based on statistical learning (cow and ‘moo’) or redundant information (same shape encoded across vision or touch). Moreover, the PRC appears to be a hub for both bottom-up, multisensory-driven processes and top-down semantic knowledge of object features. Consistent with this idea, Fairhall & Caramazza [129] reported that ‘amodal’ or abstract object concepts may be coded in lateralized regions of the temporal and prefrontal cortex. Although evidence from neurophysiological studies also supports a role for these functional regions (e.g. [130]), there are some inconsistencies in that category learning appears to be unaffected by bilateral damage to prefrontal cortices, at least in monkeys [131], suggesting that categorization may depend more on temporal regions of the brain. A further complexity may relate to the stimulus-specific features of objects: i.e. different regions within the temporal lobe are activated to different categories of familiar objects (such as animals or tools; [132]) or activation may be dependent on object function. For example, Noppeney *et al*. [133] reported that tools specifically activated regions within the temporal lobe based on their sensory features (e.g. image or word sounds) but also activated regions within the temporo-parietal lobe based on semantic knowledge of tool use. Indeed, sensorimotor interactions have previously been highlighted as an important source of information in categorization (e.g. [86]), including during development [134], although a discussion of the role of action in category formation is beyond the scope of this current review (for further information see e.g. [135]).

## 4. How do developmental processes across the lifespan affect multisensory object categorization?

Apart from incidental changes in the reliability of object information in the environment, such as the occlusion of an object's visual features or the intensity of its sound, other more long-term changes may also affect the formation of object categories. Indeed, the way in which object features, such as size or depth, are integrated across modalities can differ from childhood to older age due to changes in sensory maturation, motor control, the physical size of the body and the integrity of underlying sensory organs. For example, developmental processes are associated with the maturation of the peripheral and central nervous systems over time, as well as the emergence of category-specific processing within the ventral stream [136,137]. By contrast, sensory inputs may be less reliable in older adults due to age-related changes in the peripheral sensory organs. Consequently object recognition in older adults may depend more on prior knowledge, including multisensory object properties, acquired over the lifespan. However, ageing can be associated with a decline in both sensory function and in neural systems supporting memory, particularly the MTL (medial temporal lobe) and ATL regions of the brain. Given the significant changes in sensation and cognition that occur across the lifespan [105,138], it is reasonable to assume that development and ageing differentially affect object categorization. As a consequence, models of category formation require considerable flexibility to account for the normal fluctuations to sensory-driven, perceptual and predictive processes across the lifespan. Below we consider some of the recent findings on the role of development and ageing in multisensory processes in particular, and object categorization in general, and highlight current gaps in our understanding of the mechanisms supporting the formation of multisensory categories at each stage.

### (a) The role of development in multisensory perception and categorization of objects

Each sensory system is under a continuous process of refinement throughout the course of development, with maturation of the senses occurring at differing rates. In addition, sensorimotor exploration patterns, from grasping to crawling and beyond, become more efficient with development and can directly support cognition [139]. These factors are likely to play a role not only in the ability to perceive objects based on their multisensory features but also in forming multisensory object categories in memory [140]. Investigations into multisensory perception in infants have found evidence for their capacity to integrate sensory information [141,142] as well as map crossmodal associations [143] to form basic object categories [144,145]. However, optimal integration emerges late in development, i.e. at around age 8 or older [146–151], possibly depending on the task [152]. Prior to that, it is thought that a child's explorations of the world engage a process known as cross-sensory calibration, which is important for supporting multisensory interactions. As such, information in one sense acts to calibrate the (e.g. spatial or temporal) processing of object information in another sense, which is distinct from information across two senses being fused through inferential or Bayesian processes [153,154]. Thus, during early development perceptual judgements are guided either by the most accurate sense, or by the one with better precision, for the task (e.g. [46,155,156])^1^.

In addition, the emergence of sensitivity to temporal and spatial stimulation across the senses during development may underpin efficient multisensory perception. For example, enhanced proficiency in integrating multisensory information in 4- to 6-year-olds is associated with a narrowing of the temporal binding window across modalities [157], which provides a precise cue for shared causality [158]. Audio-visual perception of temporal synchrony has been detected in infants at four months [159] but precision improves further across development [51,160]. Other studies have investigated the role of multiple senses in spatial processing, with evidence for multisensory enhancement relative to unimodal inputs from the age of 7 years, provided (unisensory) cue reliability can be established [161] and cognitive load is minimized [162]. Overall, the evidence from studies on temporal and spatial processing suggests that multisensory perception in children, when it occurs, can be based on similar mechanisms to that in adults. For example, Verhaar *et al*. [163] reported that causal inference best explained performance in a visuo-tactile localization task in both children and adults, despite differences in perceptual bias across age groups (see also [164]).

Overall, early development is associated with rapid changes and improved precision in perceptual function across the modalities, in a process known as ‘perceptual narrowing’. Perceptual narrowing involves the enhanced perception of familiar categories, such as objects, faces and native speech, and a concomitant decline in the perception of unfamiliar categories [165]. This perceptual ability for improved discriminability between learned relative to novel stimuli early in development appears to support subsequent cognitive functions, including categorization [166]. While some evidence suggests that the categorization of objects emerges early in development, at least in the visual domain [167,168], others suggest that the development of category specificity (e.g. the processing of different categories such as faces or objects) within the visual system is more protracted [169], consistent with improved perceptual precision with development. Other evidence suggests that young children prioritize auditory over visual information from multisensory stimuli [170]. This has been attributed to differences in the rate of development across the senses [171], with a shift to adult-like visual dominance occurring only after the age of 9 years [152].

From 4 years of age, the ability to recognize ‘family resemblances’ between category members to perform category judgements is observed [172]. These effects are similar to the benefits of semantic congruency across modalities in adults [173]. Indeed, infants aged 2 years demonstrate the ability to form semantic associations across audio-visual domains and these associations become more sophisticated as the child builds experience [174]. Consequently, it might be expected that differences in the way in which objects are categorized by children may depend not only on the information available across sensory modalities but also on access to prior knowledge about the object category. Indeed, findings from studies of multisensory object perception suggest that children younger than 8 years are less selective when associating semantic cues and readily combine incongruent or irrelevant information across the senses [164].

It has been argued that the acquisition of the taxonomic organization of categories in children is perceptually based [175], with evidence for a role for feature similarity in children's ability to form categories [176] similar to that of adults. Other evidence suggests a shift during late development between the role of prior knowledge and feature similarity in determining category membership [177]. Indeed multisensory featural cues appear to benefit categorization in children. For example, Broadbent *et al*. [178] used an incidental category learning task in which children aged 6–10 years learned familiar objects presented in auditory only, visual only and audio-visual learning conditions. They reported a multisensory benefit to categorization performance for all age groups, although categorization performance overall improved with age. A subsequent investigation into visuo-haptic interactions on novel object categorization performance in children aged 6–10 years also revealed a multisensory benefit to categorization performance, but this emerged late, i.e. in 8- to 9-year-olds [179], consistent with findings on perception. Taken together with results of other studies [180,181], maturation trends were observed across all category learning conditions, suggesting that improved sensory and perceptual precision with development likely underpins multisensory integration for categorization.

Development also plays a role in children's ability to learn implicit associations between crossmodal object features, known as crossmodal correspondences [182]. Some studies have provided evidence supporting the existence of such crossmodal correspondences in newborns, including for audio-visual object features such as pitch and object elevation ([183], although see [184]). Such patterns of statistical learning have been implicated in the formation of categories during infancy and early childhood [185], including auditory categories relevant to later language acquisition [186]. Importantly, statistical learning continues to improve throughout development across all modalities [187,188]. However, in contrast with the number of investigations into the emergence of complex cognitive skills supporting visual categorization, there have been relatively few studies of object categorization based on multisensory integration in children and future studies are needed to elucidate the links between multisensory perceptual abilities and categorization during early development.

### (b) Does ageing affect multisensory object perception and categorization?

Similar to early development, the ageing process also brings about changes in sensation and perception, which in turn may impact object categorization. Category formation in healthy ageing can be shaped by both bottom-up and top-down factors: broader cognitive functioning can determine categorization ability (e.g. working memory capacity, associative memory, attention and executive functions [189,190]), while both perceptual and attentional strategies can be used to compensate for age-related cognitive decline [191], thereby modulating performance. For example, age-related changes in sensory acuity may have a minimal effect on perception because of the specific manner in which information across the senses is combined in later life (see [192,193] for reviews). In general, perception in older adults can benefit from multisensory integration relative to unimodal inputs [194], and either a wider temporal [195–197] or spatial window of integration [198] can compensate for changes in sensory inputs at the periphery. However, while the ability to perceive objects based on bottom-up processes may be preserved in ageing due to multisensory integration, categorization performance will likely also depend on cognitive function, which may be affected by task demands such as category complexity [199–202].

In particular, there is evidence for differences in object perception between younger and older adults, due to changes in ‘bottom-up’ and/or ‘top-down’ mechanisms. With regard to bottom-up processes, older adults show reduced discrimination performance between objects with a high level of feature ambiguity compared to young adults [203,204]. While both younger and older adults fixate more frequently on more salient than less salient objects, this so-called ‘salience capture’ increases with age, suggesting age differences in bottom-up processes guiding visual object selection [205]. Although perceptual sensitivity to object structure appears well preserved both visually [206] and haptically [207] in ageing, the ability to match object properties such as shape across vision and touch becomes increasingly difficult with age ([208,209]; see also [210]). However, these differences can often be attributed to cognitive rather than sensory or perceptual factors [208] as a reduction in cognitive demands can improve older adults' performance [209].

Older adults also demonstrate an increased reliance on top-down or prior knowledge when making multisensory perceptual decisions [211]. This could reflect a greater reliance on rule-of-thumb estimates when unisensory inputs decline, an accumulation of prior knowledge over the lifespan [212] or a combination of these factors. An increased reliance on priors with age may also influence object perception. For instance, when detecting objects in naturalistic scenes, performance in older adults is influenced more by the congruency between the surrounding visual context and the target object compared to that of younger adults [213,214] (see also [215] for evidence that familiar contexts influence object memory in ageing). This increased effect of scene congruity in older adults may partly function to compensate for the reduced fidelity of bottom-up signals [216].

Changes to bottom-up and top-down processes on perceptual function can, in turn, determine how categorization is achieved in older adults. For instance, a reduction in the quality of sensory input can increase categorization difficulty [217] (see also [212] for evidence linking contrast sensitivity with object identification) and may enhance the influence of information from other, more reliable modalities for object categorization (e.g. [218]). Diaconescu *et al*. [219] reported that the benefit from exposure to multisensory compared to unisensory cues was larger for older compared to younger adults in an audio-visual semantic object classification task. Consistent with a role of top-down mechanisms in object perception, older adults rely more on prior knowledge to aid decisions about object categories. For instance, visual context can aid the speed and accuracy with which objects are categorized and this benefit for context increases with age [220,221]. Older adults show enhanced semantic categorization abilities compared to young adults, presumably reflecting the acquisition of conceptual knowledge over time [222]. For example, older adults may categorize objects based on their functional, as opposed to perceptual, similarity [223].

However, some aspects of object categorization appear to remain invariant to age effects. For example, implicit memory processes do not appear to be subject to age-related decline (see [224] for a review). Indeed, similar priming effects (i.e. improved object identification to previously perceived objects) for familiar objects have been reported for vision [225], audition [226] and touch [227] as well as in crossmodal paradigms (audio-visual, visuo-haptic [226]). These findings suggest that implicit representations of familiar objects are shared across sensory modalities and maintained in healthy ageing. Implicit priming effects for novel (i.e. unfamiliar) objects have also been reported in older adults [228,229], albeit to a lesser extent over long repetition delays, which may reflect difficulties in maintaining novel object representations in working memory [228,230]. By contrast, explicit memory for novel objects is impaired in older relative to younger adults [229].

A decline in object categorization performance in older adults is consistent with evidence for changes in brain structure and function with ageing. While young and older adults show equivalent levels of bilateral activity in posterior PRC during the visual discrimination of complex objects, the anterior PRC exhibits higher levels of bilateral activity in younger versus older adults, implying age-related functional changes to this neural region ([203]; see also [204,231]). The PRC also appears less sensitive and exhibits reduced coupling with the visual cortex in older compared to younger adults [232]. These findings are in line with broader evidence that individuals with damage to the PRC show deficits in the discrimination of objects with high but not low feature ambiguity [233] or with high semantic confusability [234]. In addition, there is evidence for more robust activation of frontal regions in older compared to younger adults during tasks involving visual object discrimination [203], object naming [235], recognition [216] and conceptual classification [236]. This evidence may be interpreted as an increased, potentially compensatory, reliance on top-down mechanisms with increasing age. For example, Sebastián *et al*. [237] found evidence that older adults recruit additional neural resources to support haptic recognition compared to young adults. Collectively, these findings suggest that older adults show different functional patterns of cortical activation during object processing compared to their younger counterparts, across multiple brain regions, in accordance with age-related fluctuations in bottom-up and top-down processing, as previously discussed. However, as empirical research probing multisensory object perception in older adults is still sparse, particularly studies of neural function in object perception, future studies are needed to provide insights into how multisensory object categorization and learning are achieved as the brain ages.

## 5. How can multisensory object representations support flexible, adaptive categorization processes?

Categorization helps the human brain to extract relevant object information in order to efficiently identify an object that may be familiar or generalize to novel exemplars of that category. Based on evidence from the multisensory perception of objects across the lifespan, the represented information that supports the formation of object categories may also be integrated and multidimensional in nature. Moreover, findings that top-down processes influence object perception [108,111] suggest that the selection of relevant and specific object information is an important mechanism underpinning the formation of adaptive or flexible categorization processes in memory. The challenge is to understand better the complex feedforward and predictive processes involved in combining information from across several sensory modalities, as well as multiple feature dimensions, that form such object categories.

Previous investigations of how novel categories are formed can provide insights into the role of bottom-up processes or the accumulation of top-down knowledge in the formation of object categories and allow us to generate some predictions on their formation from multisensory sources. For example, category formation, at least in the visual domain, has been shown to be affected by the number, type, distribution and frequency of object features, including shape. One proposal suggests that generative models for object categorization may differ qualitatively according to the number (i.e. frequency) of object exemplars available and their inter-object variability [238]. In other words, the frequency distribution of the set of objects, or object features, representing a category may also affect category learning and generalization [239]. Indeed, Carvalho *et al*. [240] reported a role for both item frequency and the distribution of these items in category learning. For example, using novel object categories, they varied the relative frequency of presentations of certain category items over other items, thus manipulating the distributional properties of the learned categories. They found differences in categorization performance depending on these distributions: broader generalization performance occurred when the stimulus distribution was either skewed (e.g. relatively more repeated presentations of some items over others from the category) or uniform (equal number of presentations across category items) compared to a normal distribution. It may be possible to build upon these results to describe the formation of object categories across multiple sensory inputs: based on the nature of the crossmodal mapping (e.g. from multiple to fewer features, or from variable to limited distributions) these interactions may determine category decision boundaries and affect categorization processes. For example, different category objects may be represented by multiple unisensory features that are broadly distributed and variable (e.g. the shapes of dogs), whereas information from other senses may be less distributed or variable (e.g. ‘barking’ sounds). The less variable information source may therefore be a more reliable cue to category membership due to its certainty. Consequently, information in one modality might simultaneously act as the ‘glue’ to associate more variable or uncertain crossmodal features with category membership whilst also providing reliable cues to demarcate the category boundary. More particularly, this would allow for flexibility in categorization: for example, if featural information in one sensory stream becomes relatively uncertain due to momentary or developmental changes, then categorization may then be based on information from the more reliable or appropriate modality. Thus, hearing a bark will help recognize the shape of a dog if visual information is compromised (i.e. if the image of the dog is partially occluded or blurry).

**Figure 1.**
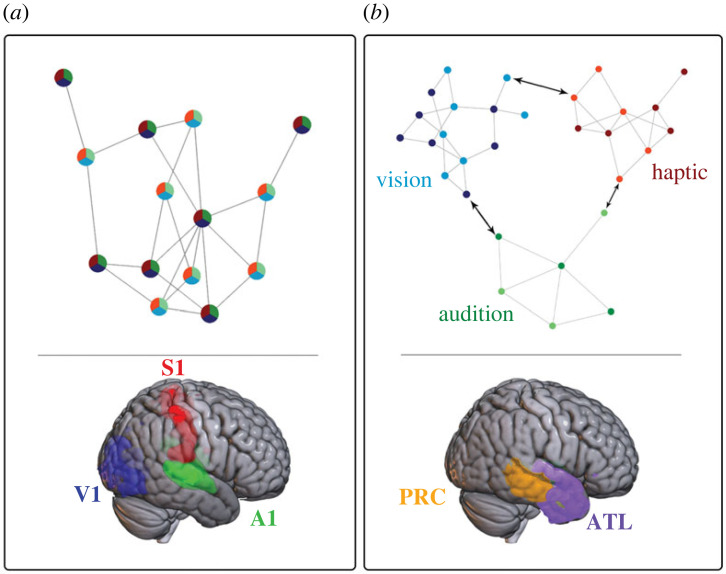
A schematic illustration of both (*a*) ‘early’ and (*b*) ‘late’ integration processes underpinning the formation of object categories. Both the feature networks (top half) and corresponding neural (lower half) loci of these mechanisms are illustrated. In (*a*), each node is a single feature that represents an integration of multisensory information, and these features provide bottom-up information that defines membership of one category (dark nodes) over another (light nodes). Multisensory integration of these features occurs early, in primary sensory regions highlighted above (V1, A1 and S1 refer to primary visual, auditory and somatosensory cortices, respectively). In (*b*), each feature is unimodal and membership of a category (dark versus light nodes) is defined by a unique combination of learned associations across these features (indicated by arrows). These crossmodal associations likely arise in later regions of the brain such as the PRC (perirhinal cortex) and ATL (anterior temporal lobe) illustrated here.

However, given the possible differences in information (not just in reliability but also frequency) across sensory modalities, it is unclear how categorization based on multisensory inputs generalizes to novel exemplars or to contextual effects such as changes in the encoded information, appropriateness of the modality, integrity of the neural system, task and goal set requiring rapid adaptation. For example, novelty may be modality specific (e.g. differences in relative frequency of information across modalities) or may be defined by the combination of learned information across modalities. This is particularly true during early development when the child is acquiring knowledge of the world. Previous reports suggest that generalization is achieved by the formation of abstract (or so-called 'amodal') categories in memory. However such a proposal does not adequately explain how generalization can occur and indeed it is intuitively unlikely, given the demands of our behavioural goals and repertoire at each developmental stage, that abstract categories in memory (e.g. [129]) could facilitate generalization to novel sensory inputs. Moreover, as discussed above, there is strong evidence for modality-specificity in object representations, with representations constrained by sensory inputs, and little neurobiological evidence supporting the existence of ‘amodal’ representations *per se* [102]. Furthermore, evidence that feedback from higher brain regions, including PRC, targets modality-specific regions of the brain [241] would be challenging to explain by models incorporating abstract representations. Instead, categories might be represented in memory based on a minimum or ‘common denominator’ set of diagnostic multisensory features that are invariant to incidental changes in the encoded information [27], such as changes in object viewpoint [242,243] or developmental fluctuations in its neural basis over the lifespan [244]. This idea is illustrated in figure 1*a* and evidence for multisensory interactions in ventral regions of the temporal lobe (discussed earlier under §3) supports this proposal. However, how category generalizability emerges from such a multisensory representational format is currently not clear without further research. Alternatively, unisensory featural representations may define a category and thus object categories may be more distributed across modalities. Specifically, object categories may be structured based on multidimensional, feature-specific spaces that are further constrained by modality-dependent processes (figure 1*b*). This idea has some support from research in neurophysiology: as discussed by Hoffman & Logothetis [245], specific object categories may be represented in the brain as a distributed code across neurons that, at the same time, maintain selectivity for individual object exemplars at the individual neuronal level.

Consistent with the idea of feature spaces underpinning object representations in the brain is the recent proposal by Ayzenburg & Behrman [246] that neurons in the temporal lobe do not code for global shape *per se* but rather for individual object features, and the function of the parietal cortex is to integrate the features into an object depending on task demands. Indeed, Rohe & Noppeney [247] reported evidence supporting this function of the parietal cortex based on the integration of multisensory features. If true, then it makes intuitive sense that object-relevant features from any sensory system are also represented in the ventral pathway and that integrated multisensory features can form the basis of categorical organization and object categories, possibly underpinned via interactions in parietal cortex. Future research is required to investigate the specific format of object representations in the brain based on multiple sources of sensory information, and how distinct categories are formed based on these multisensory distributions of features that allow for both the recognition of familiar objects and generalization to novel exemplars.

### (a) The time course of multisensory interactions and the formation of categories

Although previous neuroimaging studies highlight candidate mechanisms for supporting object perception, how and when multisensory interactions arise in the brain to affect categorical decisions may be dependent on the nature of these interactions and the stimuli themselves. On the one hand, redundant featural information may be integrated during early perceptual processes that subsequently enhance multisensory categories in memory such that categorization is facilitated (even to unisensory inputs). On the other hand, the integration of featural information across modalities may depend on learned semantic associations in later memory structures. The time course of these unimodal or multisensory (audio-visual) mechanisms in object perception was investigated by Vercillo *et al*. [248] using EEG. While early event-related potentials (ERPs); i.e. 80–100 ms post stimulus) were greater to multisensory stimuli than (the sum of) unimodal elicited ERPs, sensitivity to learned over novel objects, irrespective of modality, was observed only in later ERPs (250–300 ms). Indeed intermediate ERPs (100–200 ms post stimulus) localized over LOC also appeared sensitive to multisensory versus unisensory inputs, suggesting effects on object perception. Their finding further supports the distinction between early and late mechanisms underpinning object categorization and decisional processes (see also [249]). Moreover, evidence for late interactions is consistent with other studies suggesting greater sensitivity in ATL and frontal regions following object learning. Indeed, neuroimaging studies typically report a decrease in activation in regions within ATL with increasing familiarity of either visual [250] or tactile [251] object presentations. Moreover, Folstein *et al*. [252] suggest that object learning may even result in the emergence of relevant object features or dimensions that optimize object discriminability (see also [253]). Further evidence from animal models suggests that following exposure to objects, the PRC plays a specific role in representing multisensory object features and less of a role in modality-specific representations [254]. This finding suggests that the processing of multisensory object information may be to dynamically update object representations within the PRC based on either stimulus-dependent object properties (i.e. multisensory object representations) or the optimal processing of object information, depending on task goals (see [255] for a review). Our work on perceptual learning in the tactile domain also supports this finding: learning was associated with increased activation in the middle occipital lobe but decreased activation in brain regions supporting cognitive control, specifically frontal and parietal regions [256]. Despite these important insights, as well as those from recent studies on multisensory object perception, it remains unclear when multisensory information informs categorization. At least two possibilities come to mind, each based on whether features are coded as integrated or associated across modalities, which we discuss below and are illustrated in figure 1.

### (b) Early integration mechanisms and the formation of categories

Given the evidence for multisensory integration of object features underpinning perception, it is likely that this process also supports categorization. Yet how relevant features are extracted from a multidimensional, multisensory feature space for the purpose of efficiently categorizing an object remains unclear. Previous investigations have demonstrated category learning of stimuli along single-feature dimensional spaces, such as colour or brightness [98,257], or pitch [258]. More crucially, evidence for category boundaries has been reported for complex stimuli such as cars [259], familiar objects [260], the sex of faces [261], facial expressions [262] and speech sounds [263], which vary along integrated, multidimensional feature spaces (i.e. in which specific feature dimensions are difficult to identify). Although these studies demonstrate categorization based on unimodal features, the results may be extended to provide a description of how categorization applies to multisensory featural dimensions. Thus, if categorical learning increases the distinctiveness of items based on the binding of features within a modality, then such a process might also apply to all integrated features, including those across modalities. As outlined above, this idea is presented in figure 1*a* and can be further illustrated as follows: if we imagine the multisensory featural space characterizing all possible liquids, we can assume that some of those features may be correlated across modalities. For example, the texture of a liquid may be perceived by sight, touch (and possibly sound as it is being poured) and since texture features are correlated, then learning to categorize liquids based on texture cues through vision alone will likely benefit tactile categorization. Furthermore, the categorization task may be based on previously learned information (such as ‘tea’ versus ‘coffee’). Accordingly, prior knowledge may directly influence the selection of features that are multisensory in nature even if unimodal information is provided [264–266], i.e. certain features may be perceived as equivalent, and thus readily transferable, across modalities, such as texture. Thus, category learning along one feature dimension should ‘sensitize’ all other associated or integrated feature dimensions, even those across modalities [267]. Using the example above, liquid texture should help distinguish a café espresso from Darjeeling tea, whether perceived through vision or touch, as it is an integrated multisensory feature. In turn, prior knowledge may affect greater distinctions or increase similarity between the multisensory featural representations underpinning each category.

### (c) Late integration mechanisms and the formation of categories

A second possibility suggests that modality-specific object information is preserved in memory and that learning allows for the extraction of unimodal, low-dimensional diagnostic feature spaces in which the individual objects are maximally distinct [268]. Accordingly, the brain would maintain the ability to ‘selectively sensitize’ local regions of a dimension [98] or unisensory dimensions that facilitate task performance. For example, the sounds of ‘barks’ and ‘meows’ may be more informative than tactile shape for categorizing cats from dogs, although all such features can be relevant to the task. This idea is illustrated in figure 1*b,* in which separate feature spaces are illustrated for each sensory modality. Thus, during category learning, the brain selects feature dimensions that are maximally diagnostic of category membership, possibly based on attentional mechanisms [269]. The effect of this learning is that discriminability may be maximized for object features lying along a single, unimodal dimension that cross a category boundary, but no such direct effect on other incidental features would necessarily be observed. Instead, associations between features across modalities may be the preserve of later memory processes, although modality-specific featural representations are maintained. Studies on visual categorization that have used stimuli defined along two or more dimensions provide some evidence in support of this proposal [270]. Furthermore, evidence for flexible crossmodal interactions has also been reported. For example, Williams *et al*. [218] found that categorization of ambiguous visual images was biased by the specific nature of semantically relevant sounds. Thus, associations across modality-specific featural representations maintain category flexibility and play an important role in object categorization.

### (d) A possible role for both early and late multisensory integration processes on the formation of adaptive categories

In order for categorization to be flexible and adaptable to changing stimulus contexts, task goals or developmental stages, both early and late integration may be required to support efficient category decisions and indeed it is unlikely that these processes are mutually exclusive. For example, category formation may depend on the relative reliability of the sensory information, irrespective of modality, to dominate categorical decisions or, on the other hand, the integration of (redundant) information may help to sharpen sensitivity to categorical membership better than unimodal information alone. Furthermore, the integration of features across modalities may contribute to object perception which, in turn, forms the basis for categorization. Thus there may be a hierarchically organized system in which early interactions can be further refined by feedback from higher levels at each stage of processing. The model depicted in figure 1*a* may depict early sensory integration of correlated features, but higher levels may affect the selection of distinct features for perception or categorization through attentional mechanisms or predictive coding.

The idea for integrated or distinct featural representations underpinning category formation has historical precedence in the literature on categorization. For example, some of the earliest studies on the role of single over integrated features on speeded classification were conducted by Garner in the 1970s [271], and these findings can provide some insights into how multisensory categories may be formed. For example, Gottwald & Garner [272] referred to different levels of categorization processing depending on the number of relevant feature dimensions (from one to two in their studies) and referred to processes of ‘filtering’ when only one dimension was relevant or ‘condensation’ when more than one dimension was relevant. Speeded classification was facilitated in the condensation condition when the features along two visual dimensions were integrated (e.g. value and chroma or saturation and brightness) rather than separable (e.g. value and size or brightness and size). The opposite effect was reported for the filtering process, which was always faster but only when the dimensions were separable, not integrated. Similarly, the within-modal dimensions of auditory pitch and intensity (loudness) are also perceived as integral, with better behavioural performance on a classification task to redundant variation of these stimuli over orthogonal variation [271]. In addition, better performance to integral dimensions of pitch and loudness was reported over brightness and volume. Grau & Kemler Nelson [273] argued for a continuum of integrality from dimensions that are difficult to tease apart to separable dimensions that are easily identifiable. In addition, Goldstone [98] reported acquired distinctiveness effects on CP to stimuli in which feature dimensions were integrated rather than separable. Indeed, category learning should enhance processes underpinning category membership while also improving the discriminability of objects along the relevant dimensions. Such evidence for improved discriminability has been reported for items on one relevant dimension provided the dimensions were separable [267,274]. More recent studies have extended this work to account for crossmodal interactions. For example, Brunel *et al*. [275] reported that visual categorization performance of geometric shapes was facilitated when paired sounds corresponded to a relevant visual property (i.e. high-frequency sound with a bright shape). Furthermore, the presence of a sound during learning improved subsequent categorization performance and facilitated generalization to other category members not previously learned with a sound [276], consistent with the sensitization of relevant dimensions for object categorization.

Previous research on semantic memory has provided evidence in support of categorical distinctions based on learned associations across modalities [105,277]; however, some findings suggest that the way sensory information affects category selectivity may be flexible. For example, Skipper *et al*. [278] reported evidence to suggest that distinct sub-regions within the ATLs are organized according to both sensory-specific and multisensory information: auditory-only, visual-only and audio-visual representations activated superior, inferior and polar regions of the ATL, respectively. Moreover, temporopolar dysfunction has been linked with a breakdown in the distinction between category boundaries [279], suggesting that this region may play an important role in bridging semantic knowledge and object perception. Taken together with evidence discussed earlier, these studies suggest a flexible mechanism by which efficient categorization can be achieved when objects are defined by features along multiple, multisensory dimensions. As such, categorization may be facilitated by integrated, or bound, features across modalities (e.g. features that are spatially or temporally aligned, or statistically learned associations) relative to features defined by separate dimensions (i.e. not aligned or learned). Furthermore, given the complexity of object categories based on multiple sensory inputs, including the number and type of features as well as their underlying dimensions, it is clear that there is a role for attentional processing in the selection of the most diagnostic features or dimensions [280].

## 6. Conclusion

The brain's ability to organize sensory information into categories allows us to efficiently recognize and interact with objects throughout our lifespan. This is a remarkable achievement given the spatial and temporal complexities of our multisensory world, yet this process underpins even the most mundane of tasks. For many of us, our day may start with drinking coffee. Depending on whether you prefer an espresso or cappuccino, this daily task relies on complex perceptual–cognitive processing such as first categorizing cups from other crockery in the cupboard, then further categorizing espresso from cappuccino cups, to recognizing your favourite cup based on its relevant features such as its shape, thermal conductance, weight and size. The next steps may involve smelling the aroma of freshly ground coffee beans followed by the sound of the coffee percolating in the ‘Moka’ pot. Each step in this process involves an interaction between sensory inputs and top-down knowledge (e.g. semantic knowledge relating to coffee) as well as other cognitive processes such as attention and task maintenance. Although recent years have seen a rapid increase in our knowledge of the behavioural and brain bases of object perception from multiple sensory inputs, suggesting early cross-sensory interactions to support efficient decisional processes, a gap remains in our knowledge of how these representations are linked to support the formation of categories in memory. To date, research on object perception and categorization has proceeded in a piecemeal fashion, with parallel investigations into perceptual, higher-order cognitive and attentional mechanisms supporting our ability to recognize the content of our world. A more coherent approach to research in the field is now needed to help gain a better understanding of the neurocognitive steps leading to everyday perceptual decisions about objects, such as recognizing our own cup of coffee on the kitchen table, and how these categorization processes are affected across the lifespan.

## Data Availability

This article has no additional data.

## Endnote

1 Accuracy is the distance between perceptual and physical reality (e.g. the perceived versus actual length of a contour), whereas precision is a measure of response variability (i.e. the replicability of intra- or inter-participant responses; see [154]).
